# Double-Function Oxygen Scavenger and Aromatic Food Packaging Films Based on LDPE/Polybutadiene and Peanut Aroma

**DOI:** 10.3390/polym13081310

**Published:** 2021-04-16

**Authors:** Adriana Juan-Polo, Salvador E. Maestre Pérez, María Monedero Prieto, Ana María Tone, Carmen Sánchez Reig, Ana Beltrán Sanahuja

**Affiliations:** 1 Analytical Chemistry, Nutrition and Food Science Department, University of Alicante, P.O. Box 99, 03080 Alicante, Spain; adriana.juan@ua.es (A.J.-P.); salvador.maestre@ua.es (S.E.M.P.); 2 Packaging, Transport & Logistics Research Center (ITENE), Albert Einstein 1, Valencia, 46980 Paterna, Spain; maria.monedero@itene.com (M.M.P.); anamaria.tone@itene.com (A.M.T.); csanchez@itene.com (C.S.R.)

**Keywords:** active food packaging, oxygen scavenger, aromatic food packaging, nuts, polybutadiene, low density polyethylene, peanut aroma

## Abstract

The aim of this study was to develop a double function active packaging material for nuts. The packaging solution, on the one hand, integrated polybutadiene (PB) as an oxygen scavenger and, on the other hand, it incorporated peanut aroma (PA) to improve customer’s sensorial experience. Different formulations based on low density polyethylene (LDPE), commercial PA (5 wt %) and PB at two levels (5 wt % and 13 wt %) were obtained by cast film extrusion. The obtained films were compared in terms of their mechanical, structural, optical and thermal properties confirming a plasticizing effect of PA and PB resulting in an increase in the ductility of the polymer and in a slight decrease in the thermal properties, maintaining their transparency. Regarding the oxygen capacity of the films, values of 4.4 mL and 2.7 mL O_2_ g^−1^ film were obtained for PE/PA/PB13 and PE/PA/PB5, respectively, after 6 days proving the suitability of the UV irradiation treatment in improving the oxygen absorption capacity of PB without the need of a metal catalyst. The aroma retention capacity into the polymer matrix was also evaluated in the developed formulations. The incorporation of PB in 13 wt % into a LDPE matrix improved the PA retention. This behavior was attributed to the ability of PB in enhancing cross-linking of LDPE as the concentration of PB increases. The results suggested the potential of PE/PB/PB13 films as oxygen scavenger and aromatic food packaging system to offer protection against lipid oxidation in nuts.

## 1. Introduction

One of the main problems of the nut industry is the reduction in the shelf-life and quality of food samples, specifically for those who have a high unsaturated fat content, due to oxidation reactions that lead to a deterioration of the flavor and aroma, color alteration and nutritional losses [1]. In fact, unsaturated fatty acids are substances quite sensitive to oxidation, but they are also essential in the diet since they play an important role against cardiovascular diseases [2]. Additionally, oxidation reactions produce potentially harmful substances, such as hydroperoxides or aldehydes and ketones, as primary and secondary oxidation products, with the consequent repercussion on the consumer’s health [3].

Currently, to improve the shelf life of nut products, food industry uses different approaches, such as the addition of preservatives directly to foods. However, the use of additives is becoming less and less accepted by consumers concerned about the adverse effects they could cause, although their safety in terms of the frequency of consumption and the maximum allowable dietary level avoiding the generation of any adverse effect is always under investigation [4]. Modified atmosphere packaging (MAP) is another widely used alternative to increase the shelf life of packaged nuts [5]. However, the residual oxygen concentration in the package often remains between 0.5% and 5% despite using MAP and it can increase during storage [6] since oxygen can permeate through, even high-barrier polymeric matrices. To solve this problem, in the last years, some research studies have been focused on the development of new active packaging materials by the incorporation of an oxygen scavenger into the polymeric matrix [7]. These compounds remove oxygen from the headspace of the package thus reducing the possibility of food oxidizing, increasing its useful life, and maintaining its quality [8,9].

The use of oxygen scavengers in food packaging is implemented in different ways. One is the incorporation into the packaging of separate elements such as bags, pads, sachets or labels, which contain the oxygen scavenger system. Different systems using these formats are commercially available such as OXYCatch^TM^, FreshPax^®^ and Ageless^®^. Alternatively, oxygen scavengers can be directly integrated into the packaging materials, either in mass by using extrusion or injection processes, or into the packaging surface by way of a coating [10]. To date, the best results are obtained by metals based scavenging systems, whose reaction requires activation by moisture and their incorporation by extrusion at the industrial level is a big challenge [11].

On the other hand, polybutadiene (PB), a synthetic rubber, is a well-known oxygen absorber due to its double bonds, which are oxidized upon exposure to atmospheric oxygen at room temperature producing polyketones [12]. Despite overcoming cast extrusion issues, its low oxygen scavenging capacity has sternly hindered its uses as an oxygen absorber. Accordingly, PB has been incorporated with a transition metal catalyst to promote oxidation process [13]. In this sense, Wang et al. [14] developed an oxygen scavenging film consisting of a 1,2-polybutadiene with an iron complex by solvent casting reporting an oxygen scavenging capacity of 200 mL (oxygen gas at STP) g film^−1^. Following this line, cobalt neodecanoate has also been incorporated together with PB by using cast extrusion [15] obtaining a scavenging capacity of 90 mL (oxygen gas at STP) g film^−1^. However, the extrusion process led to unacceptable films in terms of optical and mechanical properties among others and cobalt applications are limited due to their high toxicity [16].

Recently, Kordjazi et al. [17] has described the improvement in oxygen absorption of PB due to the incorporation of TiO_2_ as a photocatalyst. However, the complexity of the extrusion process due to the presence of the catalyst have highlighted the need to develop a suitable material that can be processed by conventional processing techniques used in the packaging industry such as extrusion. Based on these results, in this work a new active packaging material by using PB as oxygen scavenger has been developed. This new material did not incorporate a catalyst but instead uses the photo-oxidation of PB when it is exposed to UV radiation [18], since UV exposure of PB accelerates vinyl bonds oxidation and increased PB oxygen absorption capacity. From the best of our knowledge, oxygen absorption activation of PB by UV-light has not been studied yet, although UV irradiation of PB has been studied since it benefits chain scission and crosslinking reactions of the diene chains [19,20].

In addition to the improvement of the packaged nut´s shelf life, the need to develop active aroma-enhancing packages has also been identified, which allows the controlled release of the aroma from the package to the nut samples [21]. In this way, it is possible to differentiate the product from the competitors and offer new sensory experiences to the consumer. Numerous studies have been carried out to develop new food packaging materials capable of retaining an aroma in the polymeric matrix and controlling its release into the food, which is not easy in many cases due to the high volatility of the chemical compounds, and their ease of diffusion within polymeric matrices [22,23]. However, pyrazines, the major flavor compounds present in roasted peanut aroma, are relatively low volatile compounds able to be successfully incorporated into polymer matrices [24].

Thus, the aim of this work is the development of a double-function active packaging material for nuts. The packaging solution, on the one hand, integrates PB as an oxygen scavenger and, on the other hand, it incorporates peanut aroma to improve customer’s sensorial experience. Different formulations based on low density polyethylene (LDPE), commercial peanut aroma (PA) and PB at two levels (5 wt % and 13 wt %) were obtained by cast film extrusion. The obtained films were compared in terms of their mechanical, structural, optical and thermal properties and their oxygen scavenger capacity. The aroma retention capacity into the polymer matrix was also evaluated in the different developed formulations to study the impact of the PB concentration.

## 2. Materials and Methods

### 2.1. Materials

Low density polyethylene (LDPE) ALCUDIA^®^ 2221FG was purchased from Repsol (Madrid, Spain) and polybutadiene (27% trans, 72% cis and 1% vinyl content, molecular weight Mn = 1530–2070) was supplied by Sigma–Aldrich Inc. (St. Louis, MO, USA). *N*-hexane (99%, gas chromatography (GC) grade) was purchased from Panreac (Barcelona, Spain) and peanut aroma was provided by Givaudan Ibérica S.A. (Barcelona, Spain).

### 2.2. Films Preparation

LDPE-based films with PB and PA were obtained by cast film extrusion. Both compounds were melt-mixed with LDPE using a Brabender DE 20/40D corotating twin-screw extruder (Plastograph, Dusseldorf, Germany). Mixing was done in a batch internal mixer Brabender in 40 rpm at five temperature stages (temperatures of 185, 180, 175, 170 and 170 °C, respectively). PB was incorporated at 5 wt % and 13 wt %. PA was added directly to the melted LDPE matrix at a fixed concentration of 5 wt %. LDPE without any additive was also prepared as a control sample. The film samples obtained were vacuum packaged in aluminum/LDPE bags and stored at −20 °C until the moment of analysis. The thickness of every sample was individually measured at room temperature at five random positions using an ID-S112B Digimatic Micrometer (Mitutoyo, Japan) obtaining average thickness values of around 70 µm.

### 2.3. Films Characterization

#### 2.3.1. Optical Properties

The UV–VIS spectra were recorded by using a spectrophotometer JASCO UV 630 (Madrid, Spain) at wavelengths from 200 to 700 nm in the transmittance (%) photometric mode. The transparency values were then calculated according to Simona and coworkers [25] as follows:

Transparency value = logT600/x(1)

where T600 is the transmittance (%) at 600 nm and x is the thickness of film samples (mm).

#### 2.3.2. Attenuated Total Reflectance-Fourier Transform Infrared Spectroscopy (ATR-FTIR)

Attenuated total reflection Fourier transform infrared (ATR-FTIR) spectra of the films were registered using a JASCO FT/IR-4700 instrument (JASCO Corporation, Tokyo, Japan) equipped with a deuterated triglycine sulphate (DTGS) detector. The measurements were conducted in the absorbance mode at 4 cm^−1^ nominal resolution using 16 scans. The region from 500 to 4000 cm^−1^ was scanned for each spectrum, and spectra were corrected against the background spectrum of air. Films were directly placed on the ATR crystal area (2 mm diameter). Three spectra replicates were registered for each sample.

#### 2.3.3. Mechanical Properties

Tensile strength (TS), elongation at break (EB) and Young’s modulus (YM) of the films were measured at room temperature by using a Testometric machine (M350–20 CT, Testometric Co. Ltd., Lancashire, UK). Tests were performed in rectangular probes (10 cm length × 1.5 cm width) and crosshead speed of 200 mm min^−1^. Before testing, all samples were equilibrated for 16 h at 50% RH. Tensile strength, elongation at break and elastic modulus were determined following the ISO 527−3:2018 standard [26]. Five repetitions were performed for each formulation

#### 2.3.4. Thermal Analysis

Differential scanning calorimetry (DSC) tests were performed using a TA Instruments Q2000 equipment (New Castle, DE, USA) under N_2_ atmosphere (50 mL min^−1^). Films (5.0 mg) were introduced in 70 µL aluminum pans and they were submitted to the following thermal program: Heating from 25 to 115 °C (3 min hold), cooling to −90 °C (5 min hold) and heating to 150 °C, all steps at 10 °C min^−1^. Thermal parameters were determined from the second heating scan. Crystallization and melting temperatures (T_c_ and T_m_) were determined at peak temperatures of the corresponding transitions, while the crystallization and fusion enthalpies (∆H_c_ and ∆H_m_) were calculated from the area of the corresponding peaks. The crystallization content (*X*_c_) of LDPE was calculated using the expression according to the following equation using the first DSC heating cycle to express the thermal history because of film processing [17]:

*X*_c_ = (ΔH_m_/ΔH°_m_W_LDPE_) * 100 (2)

where ΔH_m_ is the melting enthalpy per unit mass of the sample, ΔH^°^_m_ the theoretical value of the melting enthalpy per unit mass of 100% crystalline PE (293 J/g) [27] and W_LDPE_ is the weight fraction of LDPE matrix.

Thermogravimetric analyses were carried out by using TGA/SDTA851e/SF/1100 Mettler Toledo (Schwarzenbach, Switzerland) equipment. Films (4.0 mg) were heated from 30 to 700 °C at 10 °C min^−1^ under N_2_ atmosphere (50 mL min^−1^). The initial degradation temperature (T_ini_) calculated at 5% of weight loss and the temperature of maximum decomposition rate (T_max_) were determined. All analysis described above were performed in triplicate.

#### 2.3.5. Oxygen Absorption Capacity Measured after a Previous UV Activation of the Films

In a preliminary study, 0.2 g of each film were put into a quartz spectrophomoter cell 6/GL14 with septum (Starna Cientific, Ltd.; UK), afterwards they were placed into a photo light box containing a UV lamp (TUVPLS, Philips Amsterdam, The Netherlands) working at 254 nm for their irradiation. Samples were placed at 22 cm under the UV lamp. The oxygen contents inside the cell were measured after 5, 10 and 20 h of exposure by a headspace O_2_/CO_2_ analyzer OXYBABY^®^ 6.0 (WITT-Gasetechnik GmbH and Co KG, Witten, Germany). To evaluate the mechanical properties of the films after 20 h of UV irradiation, TS, EB and YM of the films were measured as described in Section 2.3.3. Due to PE/PA/PB5 and PE/PA/PB13 films showed oxygen absorption capacity after irradiating them, both references were tested as discussed below.

To optimize the irradiation time, films (1 g) were irradiated during different periods (5 h, 10 h and 20 h). Afterwards, activated films were placed into a 20-mL sealed vials and kept at 30 °C in an incubator (Inkubator 100, Heidolph Instruments GmbH and CO, Schwabach, Germany). The oxygen content inside the vial was measured after 6 days of exposure by using a headspace O_2_/CO_2_ analyzer OXYBABY^®^ 6.0 (WITT-Gasetechnik GmbH and Co KG, Witten, Germany) by puncturing the probe through a foam rubber seals. Measurements were carried out in triplicate and the oxygen absorption values were expressed as mL oxygen g^−1^ film.

Finally, different amounts of film (0.25, 0.5, 0.75 and 1 g) were irradiated at the optimum irradiation time for each formulation and then, the irradiation procedure and oxygen absorption were determined as quoted previously. Measurements were carried out in triplicate and the oxygen absorption values were expressed as mL oxygen g^−1^ film. At the optimum irradiation time of each formulation and after 6 days of exposure, FTIR analysis of the films were carried out by using the same procedure described previously in Section 2.3.2.

### 2.4. Quantification of Peanut Aroma Compounds Present in PE/PB-Based Films after Processing

PA is characterized by its pyrazines and aldehydes content being 2,3-dimethylpyrazine (2,3-DMP), 2,6-dimethylpyrazine (2,6-DMP) and nonanal the main compounds present in the PA formulation. Both pyrazines and nonanal were quantified in PA by using a standard calibration curve. The calibration curve was carried out by incorporating different amounts (3.0–5.5 mg) of a diluted PA sample in *n*-hexane (1552.63 mg kg^−1^) on 20 mL vials. The compounds were determined by headspace-solid-phase microextraction (HS-SPME) followed by GC–MS analysis by using an Agilent 6890N GC System (Palo Alto, CA, USA) coupled to triple quadrupole mass spectrometry (Agilent 5973N, Palo Alto, CA, USA) equipment. The automated sample preparation procedure was performed with a Gerstel MultiPurpose autosampler (TDS-2, Gerstel GmbH, Mülheim an der Ruhr, Germany). Samples were heated at 75 °C for 25 min. Afterwards, the SPME fiber (DVB/CAR/PDMS SPME, 50/30 m, StableFlex, 1 cm long) fixed on the mechanical arm was exposed to headspace of the sample vial for extraction of the compounds. After 60 min of exposure, the fiber was withdrawn and introduced into the GC injector for desorption (3 min) and analysis. GC–MS injection port was set at 250 °C in the splitless mode. A DB-624 column (30 m × 250 μm × 1.4 μm; Agilent, California, USA) was used which was programmed from 50 to 250 °C (hold 12 min) at 10 °C min^−1^. Helium was used as carrier gas (1 mL min^−1^) and samples were analyzed in triplicate.

To quantify the amount of aroma retained in the films after processing, a piece of each film (0.004 g) was placed in a 20 mL vial and sealed with an aluminum crimp cap provided with a polytetrafluroethylene/silicone septum. The sample vial was placed in the sample tray and then it was transferred by a mechanical arm of the autosampler from the sample tray to the agitator with a temperature controller. The same HS-SPME procedure described above was employed to quantify the amount of 2,3-DMP, 2,6-DMP and nonanal in film´s samples. The amount of aroma present in the films was calculated as the sum of the contents of 2,3-DMP, 2,6-DMP and nonanal. Each vial was analyzed five consecutive times to extract analytes quantitatively and the analysis was carried out in triplicate.

The retention capacity (%) of the peanut aroma in the polymer matrix was calculated as the comparison of the amount of the aroma found in the packaging material after processing (mf; g aroma g^−1^ polymer matrix) and the theoretical content of the aroma initially added to the material (m_0_; g aroma g^−1^ polymer matrix) [21,28] following this equation:

Retention capacity (%) = (m_f_/m_0_) × 100 (3)

### 2.5. Statistical Analysis

Statistical analysis of experimental data was performed with SPSS commercial software (Version 15.0, Chicago, IL, USA). A one-way analysis of variance (ANOVA) was carried out. Differences between mean values were assessed based on confidence intervals using the Tukey’s test for pairwise multiple comparisons at a *p* < 0.05 significance level.

## 3. Results and Discussion

### 3.1. Films Characterization

#### 3.1.1. Optical Properties

**Figure 1:**
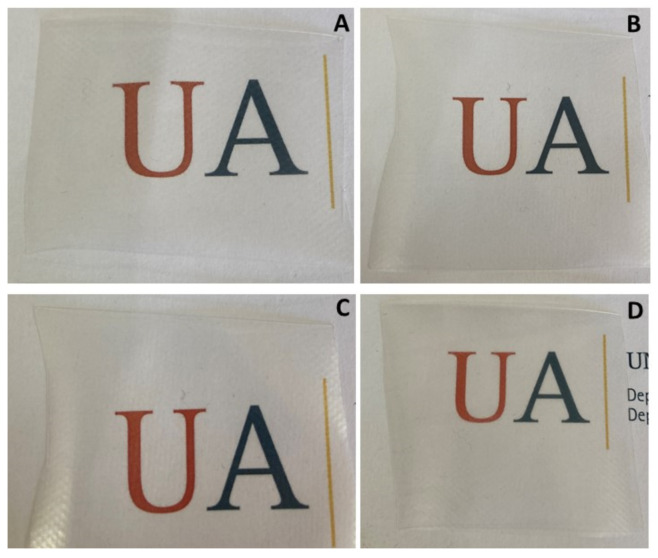
Visual appearance of neat PE (**A**), PE/PA (**B**), PE/PA/PB5 (**C**) and PE/PA/PB13 (**D**) films.

The optical properties of the developed films were evaluated since high transparency of the packaging is required in the visible region to provide consumers with a visual control of the commodity’s condition [25]. The transmittance indicates how much light can get through the material [29] and film transparency has been reported as a suitable tool to get information about the particle size of the dispersed particles [30]. Transparency values at 600 nm (t600) of all the developed films are shown in Table 1. No significant differences were found in transparency values for all the formulations, maintaining a good transparency even for PE/PA/PB13 and indicating that the LDPE/PA/PB systems were miscible blends as it can be seen in Figure 1.

The obtained results are in accordance with previous studies that confirm a high transparency of neat LDPE films that was not significantly reduced by the addition of thermoplastic starch–clay hybrids, indicating the homogeneous dispersion of particles of both additives inside the polymer matrix [30]. Following this line, Ramos et al. [31] studied the incorporation of a volatile and aromatic compound such as thymol in PLA matrices. The reported results confirmed that the incorporation of an aromatic additive at a concentration level lower than 10 wt % did not affect dramatically the transparency of PLA films.

#### 3.1.2. ATR-FTIR Analysis

**Figure 2:**
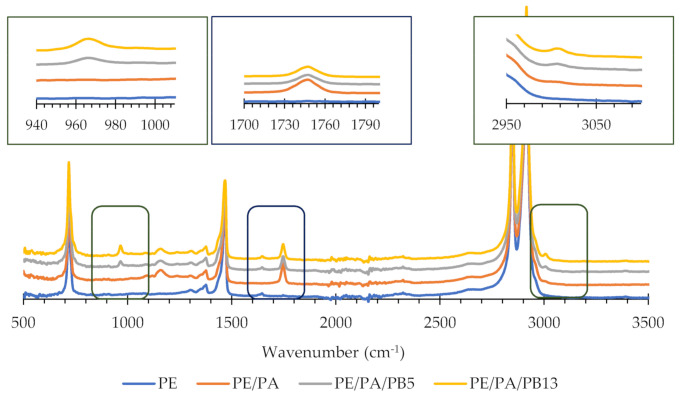
Attenuated total reflectance-Fourier transform infrared spectroscopy (ATR-FTIR) spectra of PE, PE/PA, PE/PA/PB5 and PE/PA/PB13 films.

ATR-FTIR absorption spectra of developed films are shown in Figure 2. Several absorption bands characterize the FTIR spectrum of neat PE, such as the two strong bands that appear at 2919 and 2851 cm^−1^ respectively, which can be attributed to the CH_2_ asymmetric and symmetric stretching, or the band that appears at 1463–1473 cm^−1^ that is assigned to CH_2_ bending. In addition, a band appears at 720–731 cm^−1^ corresponding to the vibrational band of the rocking of CH_2_ groups [32].

The presence of PA after film processing was confirmed in the three formulated films according to the absorption peak at 1745 cm^−1^, which represented the stretching vibration of ester carbonyl (C=O) of the triglycerides (see zoomed area in Figure 1) being a clear indication of the presence of this additive in the processed formulations [33]. On the other hand, when PB was include in the formulations an absorption band at 3007 cm^−1^ appeared which corresponded to olefinic C–H stretching, in addition a signal at about 968 cm^−1^, which is assigned to trans 1,4 units [34] was also present. These results revealed a good dispersion and distribution of PB in the extruded films because of a physically stable blend.

#### 3.1.3. Mechanical Properties

The mechanical properties of the developed films were evaluated since the film integrity is a crucial requirement in food packaging applications. When compared to neat LDPE, no significant differences (*p* < 0.05) in elongation at break and tensile strength were observed with PA incorporation at 5 wt % and PB at 5 and 13 wt % (Table 2). However, the addition of PA and PB caused a decreased in Young´s modulus values. This effect could be explained as a plasticizing effect of PA and PB resulting in an increase in the ductility of the polymer. These results are in accordance with the ones obtained by other authors in LDPE films containing a volatile liquid aromatic compound such as carvacrol where a marked decrease in elastic modulus was noticed due to the plasticizing effect of carvacrol [35]. In this sense, it is important to highlight that the physical state of the PA, i.e., a liquid, promotes a better intimation of the additive into the polymer chains permitting the enhancement of the internal movement and consequently improving its ductile properties as reported previously [36]. In relation to the addition of different amounts of PB to polyethylene, it has been reported that an increase in elasticity is observed as the amount of PB increases, acting the PB in a similar way to that of a plasticizer [37].

#### 3.1.4. Thermal Characterization

TGA and DSC tests were carried out to study the influence of the addition of PA and PB on the thermal stability and crystallinity of LDPE-based films. All samples presented a characteristic DSC curve showing exothermic crystallization and endothermic melting transitions at around 98 °C and 108 °C, respectively. Melting and crystallization temperatures and enthalpies and calculated crystalline contents for LDPE samples are summarized in Table 2.

As it is shown, the addition of PA and PB to the LDPE did not modify the melting temperature (T_m_). However, a significant change in the melting enthalpy was detected, which corresponded to the decrease in the weight fraction of LDPE in the copolymer because of incorporation of the additives. Similar results were obtained by Beg et al. [38] related to LDPE/thermoplastic starch composites. Regarding crystallization content (*X*_c_), this parameter slightly increased with the addition of PB (*p* < 0.05) due to the interaction between PB and the polymer matrix showing some plasticizing effect as indicated previously by the mechanical properties. Persico et al. [35] reported similar results working with LDPE denoting that the increase in the chain mobility promoted by the plasticizing effect enhanced the ability of the polymer to crystallize.

Regarding TGA, the initial degradation temperatures (T_ini_) determined at 5% weight loss and the maximum degradation temperatures (T_max_) of the studied films are reported in Table 2. Developed films showed one degradation step with a T_max_ around 475 °C. No statistically significant differences in T_max_ were observed among all the studied samples. However, the incorporation of both additives, PA and PB at different concentrations led to a significant reduction on T_ini_ values of the films (*p* < 0.05). In this sense, when compared to neat LDPE, the addition of 5 wt % of PA, resulted in a 15 °C reduction in T_ini_ whereas the addition of 5 wt % and 13 wt % of PB confirmed a decrease in T_ini_ of 19 and 26 °C, respectively. These results could be explained by the plasticizing effect of the additives, as already suggested by mechanical and DSC results. In this sense, it has been reported that the addition of plasticizers to different polymer matrices result in some decrease in the thermal properties [36].

### 3.2. Oxygen Absorption Capacity

**Figure 3:**
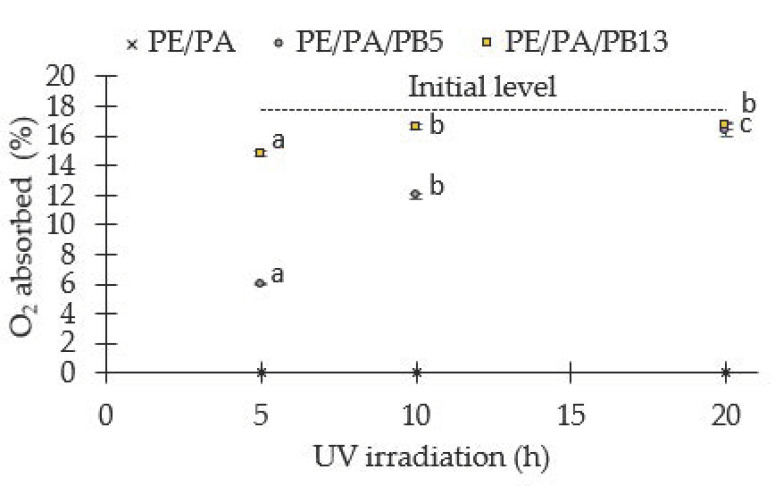
O_2_ absorbed (%) by PE/PA, PE/PA/PB5 and PE/PA/PB13 films after UV radiation for 5, 10 and 20 h (mean ± SD, *n* = 3). Different superscripts within the same formulation indicate statistically significant different values (*p* < 0.05).

The oxygen scavenging properties of the systems PE/PA, PE/PA/PB5 and PE/PA/PB13 developed by cast film extrusion were evaluated since the presence of residual oxygen in nut packages results in quick spoilage of fatty products by oxidation of fats. In a preliminary experiment, the O_2_ absorbed (%) by PE/PA, PE/PA/PB5 and PE/PA/PB13 films after UV radiation for 5, 10 and 20 h (mean ± SD, *n* = 3) was evaluated as it is shown in Figure 3. Higher values of absorbed oxygen (%) were obtained as the irradiation time and PB concentration increased. However, the PE/PA film showed no oxygen absorption regardless of the irradiation time. This indicated that the absorption capacity of the films was related to the amount of PB present in the formulation in spite that PA was also present. These results are in line with the ones reported previously by Speer and coworkers [39] confirming the oxygen absorption capacity of 1,2-polybutadiene in combination with a transition metal catalyst.

**Figure 4:**
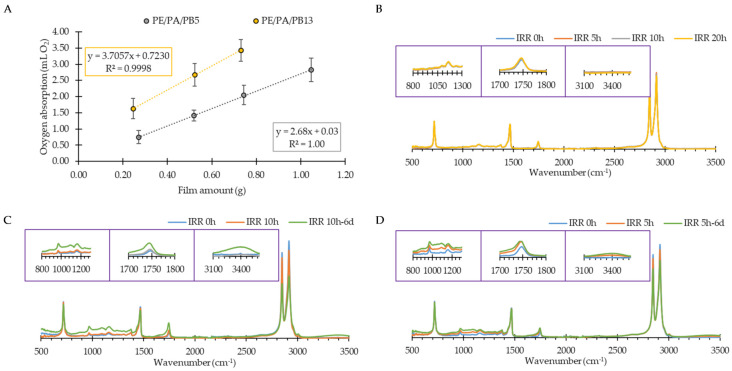
Relationship between oxygen absorption (mL O_2_ g^−1^ film) and the amount of film (g) (**A**), FTIR spectra of PE/PA (**B**) after UV irradiation for 5 (IRR 5 h), 10 (IRR 10 h) and 20 h (IRR 20 h) and of PE/PA/PB5 (**C**) and PE/PA/PB13 (**D**) films after UV irradiation for 10 and 5 h, respectively and after 6 days of oxidation (IRR10 h-6 d and IRR 5 h-6 d) in air at 30 °C.

To evaluate the impact of the UV radiation on the mechanical properties of the films, the TS (MPa), EB (%) and YM (MPa) of the films after being exposed for 20 h to UV irradiation were measured. In relation to the formulation PE/PA, values of 17 ± 1, 157 ± 13 and 120 ± 20 were obtained, respectively indicating no significant differences in comparison with the same films without having undergone UV treatment. In addition, Figure 4B shows that UV irradiation treatment produced no significant changes in FTIR spectra of PE/PA. When the PB containing films were considered, the obtained values of TS (MPa), EB (%) and YM (MPa) were 18 ± 1, 141 ± 11 and 140 ± 30 for the PE/PA/PB5 and 17 ± 2, 180 ± 35 and 151 ± 20 for the PE/PA/PB13. When comparing these data to those obtained when the films were not irradiated, it could be concluded that 20 h of UV irradiation exposure had no significant impact on the mechanical properties of the films (Table 2). In addition, the UV irradiation treatment produced no significant changes in FTIR spectra of PE/PA/PB5 and PE/PA/PB13 as shown in Figure 4C,D.

With the purpose of optimizing the UV irradiation time for each of PB containing formulations, PE/PA/PB5 and PE/PA/PB13, the oxygen absorption capacity (mL O_2_ g^−1^ film) was measured. To this end, 1 g of 5, 10 and 20 h irradiated film was used in each experiment. The absorbed O_2_ volumes were 2.1 ± 0.1, 2.7 ± 0.3 and 1.8 ± 0.2 mL O_2_ g^−1^ film for the PE/PA/PB5 films irradiated during 5, 10 and 20 h. For the PE/PA/PB13 films, values of 3.4 ± 0.3, 3.7 ± 0.2 and 3.6 ± 0.2 mL O_2_ g^−1^ film for the 5, 10 and 20 h of UV treatment were achieved. Based on these results and considering that the maximum amount of O_2_ present in the vial was 3.8 mL O_2,_ it can be concluded that 10 h of UV treatment was the optimum treatment time for the formulation with less PB content. In contrast, for the PE/PA/PB13 formulation no differences in the absorbed volume of oxygen with irradiation time were found, hence the lower irradiation time was considered as the optimum. Based on this, different amounts of films (0.25, 0.5, 0.75 and 1 g) were irradiated at the optimum UV irradiation time, obtaining the results shown in Figure 4A.

Taking as an example a packaging for 200 g of nuts, with an area of 0.0432 m^2^ and considering a 1.5% of O_2_ residual, the required absorption capacity of the film was 195 mL O_2_ m^−2^. Based on the results obtained in this study, the PE/PA/PB5 films irradiated for 10 h presented an oxygen absorption capacity of 177.5 mL O_2_ m^−2^ measured after 6 days whereas the formulation PE/PA/PB13 irradiated for 5 h achieved 222.5 mL O^2^ m^−2^, being suitable for the selected application. When comparing the obtained results with those reported previously in the literature, Kordjazi et al. [17] developed TiO_2_ catalyzed hydroxyl terminated polybutadiene (HTPB) based oxygen scavenging blends with LDPE, obtaining an oxygen absorption capacity of 30 mL O_2_ g^−1^ film after 30 days for the LDPE/PB (10 wt %)/TiO_2_ (5 wt %). However, without the incorporation of TiO_2_ as metal catalyst, after 30 days, only a value of 5 mL O_2_ g^−1^ film was achieved for LDPE/PB (10 wt %) formulation. In another study, polybutadiene films were developed by using an iron complex *N,N*′-bis(salicylidene)ethylenediamine-benzylimidazole as a catalyst and methyl linoleate [14]. The oxygen scavenging capacity of the films was up to 200 mL (oxygen gas at STP) g film^−1^ after 30 days, but the toxicity of the iron complex makes them inadequate for food packaging applications.

In the present work, a value of 3.8 mL O_2_ g^−1^ film was achieved for LDPE/PB (5 wt %) formulation after 56 days without applying the UV treatment being in accordance with the previous reported results [17]. Using the UV irradiation treatment applied in this work, values of 4.4 mL and 2.7 mL O_2_ g^−1^ film were obtained for PE/PA/PB13 and PE/PA/PB5, respectively, after 6 days (Figure 4). The results presented in this work proved the suitability of the UV irradiation treatment in improving the oxygen absorption capacity without the need of a metal catalyst, avoiding the limitations associated with the catalysts such as the reduction in transparency of the films, mixing difficulties in the extrusion process and toxicity concerns [40].

To follow the chemical changes taking place during the oxygen absorption after UV activation, film´s FTIR spectra were registered after the UV treatment and at 6 days of oxidation in air at 30 °C (Figure 4C,D). The oxidation of double bonds of PB led to a production of epoxides (bands between 800 and 1200 cm^−1^), carbonyl groups (band at 1750 cm^−1^) and hydroxyl groups (signal at 3400 cm^−1^) [14,16]. These changes were more noticeable for the PE/PA/PB13 formulation, whereas for the PE/PA/PB5 formulation these changes were less pronounced, although this film suffered from a higher UV exposure. These results support the conclusion that UV optimum activation time depended on the PB concentration with lower times needed for higher PB concentrations. In addition, higher PB concentration increased the oxygen absorption capability of the PE based films as the signal of the quoted bands continued increasing after irradiation, demonstrating the PB activation process induced by UV radiation.

### 3.3. Aroma Retention Capacity

Prior research involving roasted peanut flavor have reported that many of the substances perceived by evaluators are aldehydes and pyrazines [41]. In this sense, pyrazines are responsible for the roasted and nutty flavor in peanuts and nonanal produces a milky and toasty flavor [41,42]. Accordingly, 2,3-dimethylpyrazine (2,3-DMP), 2,6-dimethylpyrazine (2,6-DMP) and nonanal have been employed in the present work as indicators of the aroma incorporation in the developed films.

PA initial analysis indicated that 2,3-DMP, 2,6-DMP and nonanal were present at 2.44 wt %, 1.60 wt % and 0.34 wt % levels, respectively. Based on this and considering that an initial amount of 5 wt % of PA was added into the LDPE-based films, 0.12 wt % of 2,3-DMP, 0.08 wt % of 2,6-DMP and 0.02 wt % of nonanal are the maximum contents of these compounds present in the films if an incorporation of 100% is assumed.

**Figure 5:**
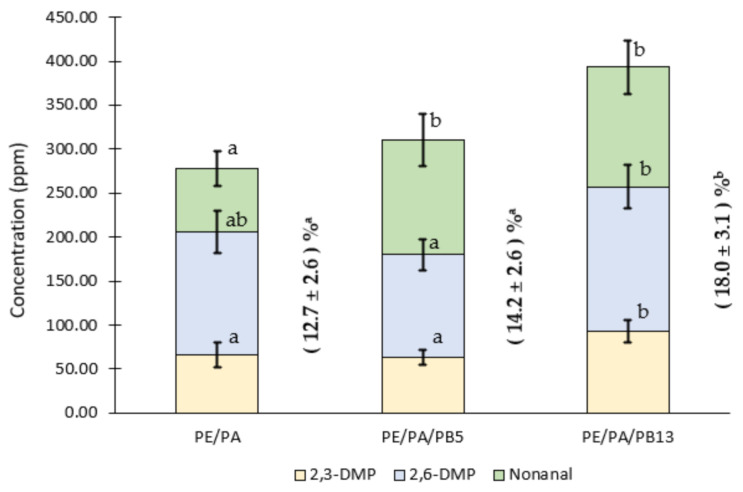
Concentration (ppm) and retention capacity (%) of 2,3-DMP, 2,6-DMP and nonanal present in PE/PB-based films after processing. Different superscripts within the same compound for the different formulations indicate statistically significant different values (*p* < 0.05). Different superscripts within the values of the sum of the individual retentions (%) of the main aroma compounds (i.e., 2,3-DMP, 2,6-DMP and nonanal) for the different formulations indicate statistically significant different values (*p* < 0.05).

The quantification of these compounds in films proved that commercial PA was successfully incorporated into PE and PE/PB based formulations. The content of 2,3-DMP found in PE/PA, PE/PA/PB5 and PE/PA/PB13 was 66 ± 14, 63 ± 9 and 93 ± 13 ppm, respectively reporting a higher significant amount of this compound in the formulation PE/PA/PB13. In relation to the 2,6-DMP, the content present in the PE/PA, PE/PA/PB5 and PE/PA/PB13 samples was 139.6 ± 23.3, 117 ± 18 and 165 ± 24 ppm, respectively. Data showed significant differences between the formulations with different wt % of PB, resulting in a higher presence of this compound in the PE/PA/PB13 formulation (Figure 5). It is important to consider that these concentrations were higher than the concentrations of pyrazines generated during the roasting process of peanuts [43], indicating that the levels of both pyrazines incorporated into the LDPE-based films were suitable to improve the customer’s sensorial experience. On the other hand, in relation to the nonanal content present in the developed films, values of 72 ± 19, 131 ± 30 and 136 ± 31 ppm were obtained in samples of PE/PA, PE/PA/PB5 and PE/PA/PB13, respectively. Significant differences were observed among PE/PA films and the ones containing PB at 5 wt % and 13 wt % indicating a positive effect of the addition of PB in the final amount of nonanal present in the films despite the high volatility of this compound.

Based on the sum of the individual retentions of the main aroma compounds (i.e., 2,3-DMP, 2,6-DMP and nonanal) no significant differences between the retention capacity of PA into PE/PA and PE/PA/PB5 films was found (12.7% ± 2.6% and 14.2% ± 2.6% respectively). However, the retention capacity of the aroma in PE/PA/PB13 films was significantly higher (18.0% ± 3.1%). Consequently, the incorporation of PB in high levels into a LDPE matrix improved the PA retention. This behavior could be attributed to the ability of PB in enhancing crosslinking of LDPE as the concentration of PB increases as reported by other authors [37]. In relation to the retention of aroma compounds in crosslinked polymer matrices, higher retention values have been reported because of the higher interactions between aroma compounds and polymer chains that occur in comparison with non-crosslinked polymers [43].

## 4. Conclusions

Results shown in this work indicate that is has been possible to obtain food packaging films with double-function, i.e., oxygen scavenging and aromatic by incorporating PB and PA in the formulations. The incorporation of PB (5 and 13 wt %) and PA (5 wt %) into LDPE by cast film extrusion slightly influenced thermal and mechanical properties of films when compared to neat LDPE. A plasticizing effect was confirmed because of the addition of PA and PB resulting in an increase in the ductility of the polymer and in a slight decrease in the thermal properties. However, all the obtained films were transparent, showing no statistical differences in relation to the optical properties in comparison with LDPE. Overall, these results indicate the technical feasibility of the developed materials for use in food packaging applications. Regarding the oxygen absorption capacity of the films, values of 4.4 mL for PE/PA/PB13 and 2.7 mL O_2_ g^−1^ film for PE/PA/PB5 formulations were obtained after 6 days. These values were achieved without the incorporation of a metal catalyst in the formulation but using an UV activation procedure. The incorporation of PB in 13 wt % into a LDPE matrix improved the PA retention, being this behavior attributed to the ability of PB in enhancing crosslinking of LDPE as the concentration of PB increased. The results suggested the potential of PE/PB/PB13 films as an oxygen scavenger and aromatic food packaging system to offer protection against lipid oxidation in nuts improving the consumer’s experience avoiding the use of additives or additional packaging elements. A scale-up of this study including industrial processing extrusion techniques would be needed to obtain commercial materials based on these formulations. In addition, further work is needed to validate the developed materials in a real scenario, such as the use of this material to pack nuts and to study its impact in nut´s shelf-life and organoleptic properties avoiding oxidation reactions and improving the customer´s sensorial experience.

## Figures and Tables

**Table 1:** Different developed formulations, their codification and transparency at 600 nm (t600) (mean ± SD, *n* = 3). Same superscripts within the same column indicate no statistically significant different values (*p* < 0.05).

Formulations	Code	t600
LDPE	PE	26.9 ± 0.3 ^a^
LDPE + 5 wt % PA	PE/PA	27.4 ± 0.2 ^a^
LDPE + 5 wt % PA + 5 wt % PB	PE/PA/PB5	27.4 ± 0.2 ^a^
LDPE + 5 wt % PA + 13 wt % PB	PE/PA/PB13	27.3 ± 0.3 ^a^

**Table 2:** The results obtained for thermal (mean ± SD, *n* = 3) and mechanical properties (mean ± SD, *n* = 5) of the studied films. Different superscripts within the same column indicate statistically significant different values (*p* < 0.05).

	YM (MPa)	EaB (%)	TS (MPa)	T_c_ (°C)	ΔH_c_ (J g^−1^)	T_m_ (°C)	ΔH_m_ (J g^−1^)	X_c_ (%)	T_ini_ (°C)	T_max_ (°C)
PE	150 ± 20 ^a^	183 ± 25 ^a^	18 ± 1 ^a^	98 ± 0 ^a^	82 ± 2 ^a^	108 ± 0 ^a^	118 ± 2 ^a^	40 ± 1 ^a^	432 ± 1 ^a^	476 ± 1 ^a^
PE/PA	110 ± 18 ^b^	199 ± 25 ^a^	17 ± 1 ^a^	98 ± 0 ^a^	78 ± 2 ^a^	108 ± 0 ^a^	111 ± 0 ^b^	40 ± 0 ^a^	417 ± 1 ^b^	475 ± 1 ^a^
PE/PA/PB5	100 ± 10 ^b^	155 ± 15 ^a^	17 ± 1 ^a^	98 ± 0 ^a^	78 ± 1 ^a^	108 ± 0 ^a^	111 ± 1 ^b^	42 ± 0 ^b^	413 ± 1 ^c^	475 ± 1 ^a^
PE/PA/PB13	110 ± 10 ^b^	170 ± 21 ^a^	16 ± 1 ^a^	98 ± 0 ^a^	78 ± 0 ^a^	108 ± 0 ^a^	108 ± 2 ^b^	45 ± 1 ^c^	406 ± 1 ^d^	475 ± 1 ^a^

## Data Availability

The data presented in this study are available on request from the corresponding author.
